# Case report: Unilateral paralysis of the hypoglossal nerve as the only clinical sign of clivus meningioma - a case report and literature review

**DOI:** 10.3389/fonc.2024.1337680

**Published:** 2024-01-24

**Authors:** Jelena Stamenović, Biljana Živadinović, Vanja Đurić

**Affiliations:** ^1^ Faculty of Medicine, University of Niš, Niš, Serbia; ^2^ Neurology Clinic, University Clinical Center Niš, Niš, Serbia; ^3^ Polyclinic “Neuromedic”, Niš, Serbia

**Keywords:** Clivus meningioma, hypoglossal nerve paralysis, dysphagia, dysarthria, case report

## Abstract

**Introduction:**

Clivus meningiomas are benign tumors that occur at the skull base in the posterior cranial fossa. Symptoms usually progress several months or years before diagnosis and may include: headache, vertigo, hearing impairment, ataxia with gait disturbances, sensory problems. In the neurological findings, paralysis of the lower cranial nerves is most often seen, which in the later course can be accompanied by cerebellar and pyramidal signs until the development of a consciousness impairment.

**Case presentation:**

We presented the case of a patient who at the time of diagnosis had only unilateral hypoglossal nerve paralysis with dysarthria and mild dysphagia. After the neurosurgical procedure, pathohistological analysis confirmed meningothelial meningioma.

**Conclusion:**

Early recognition of clivus tumors, which include meningiomas, is necessary in order to implement an adequate therapeutic procedure and prevent further deterioration of the patient’s condition.

## Introduction

When the general practitioner recognizes the clinical symptoms and signs of a neurological disorder, he will certainly refer the patient to a neurologist. However, sometimes the symptomatology is scarce, non-specific, so that the neurologist at first cannot orientate exactly which part of the nervous system is affected. At the beginning of the clinical presentation, the clinical symptoms and signs may correspond to one localization, but with further differential diagnostic procedures, it turns out to be something completely different. In order not to waste precious time and prevent enrichment of clinical symptomatology, it is necessary to determine the exact localization and character of the pathological process as soon as possible.

Pathological processes in the clival region may be initially asymptomatic or with clinical symptoms ranging from headache and cranial nerve paralysis to limb weakness with gait disturbance and altered state of consciousness. Pathological changes affecting the clivus vary from neoplasms to non-neoplastic, inflammatory or traumatic lesions. Chordomas are the most common neoplasms of the clivus, grow slowly, are locally invasive and have a high recurrence rate. Due to the notochordal origin, chordomas are midline entities and present as a midline soft tissue lesion with bone destruction. Chondrosarcoma, which accounts for 6% of skull base tumors, can involve the clivus. These tumors develop from primitive mesenchymal cells in synchondroses of the skull base. Therefore, they represent paramedial lesions that often spread not only to the clivus and sphenoid sinus but also to the middle and posterior cranial fossa. They grow slowly, can reach large dimensions, causing erosion of bones and displacement of neurovascular structures. Meningiomas are the most common primary tumors of the central nervous system and are considered to be mostly benign lesions with favorable survival rates. Some clival meningiomas are entirely dural and subdural, but others may tend to involve extradural and osseous compartments. The clivus can also be the site of metastatic tumors. In this case, the most common primary tumors are found in the kidneys, liver (hepatocellular carcinoma), prostate, thyroid gland, lungs, breast, gastrointestinal tract (adenocarcinoma), as well as lymphoma and melanoma (1)..

It is considered that meningiomas are about 30% of all brain tumors. They can be benign or malignant, diverse in intracranial locations and pathology. They are classified into three grades according to the World Health Organization (WHO) classification and 15 histological subtypes (2). Clivus meningiomas are rare and represent 5-11% of all posterior fossa meningiomas and approximately 0.15% of all intracranial neoplasms (3).

We presented a case of clivus meningioma that was manifested only by right-sided hypoglossal paralysis with mild dysarthric disturbances and difficulties in chewing and swallowing. **T**he purpose of this case report is to describe and discuss the clinical presentation of clivus meningioma.

## Case presentation

### Patient information

A male patient, 56 years old, Caucasian, referred due to difficulty in pronouncing words, chewing and mild swallowing difficulties is presented. The complaints started 7-8 months before the examination and gradually worsened. Until then, he was treated for hypertension and regularly used prescribed therapy with well-regulated blood pressure values.

### Clinical findings

**Figure 1:**
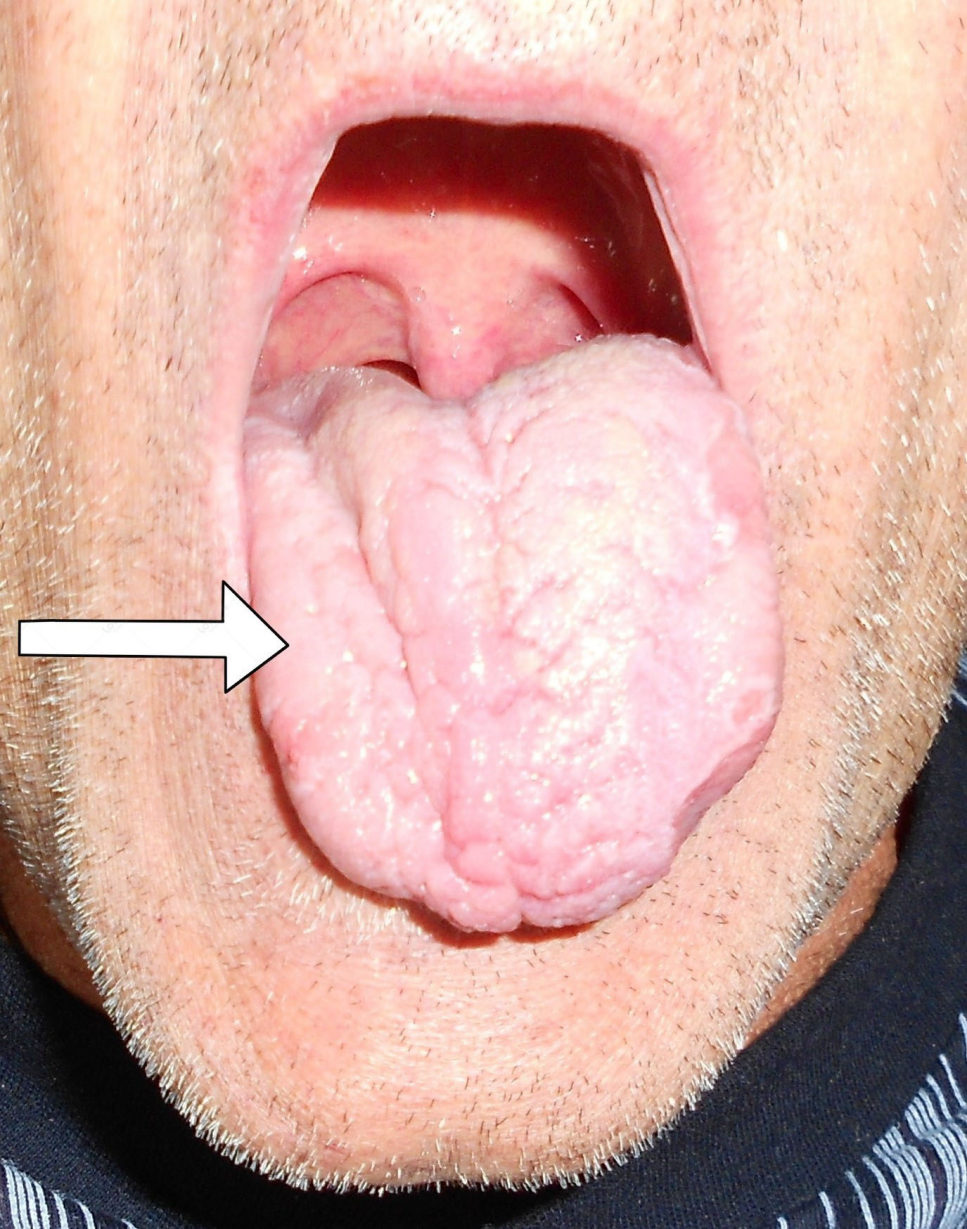
Right-sided hemiatrophy of the tongue.

A neurological examination revealed paralysis of the right hypoglossal nerve of the peripheral type with hemiatrophy of the right half of the tongue (**Figure 1**). Mild dysarthria was observed during the conversation with the patient. Findings on the other cranial nerves were normal on both sides. Examining the upper and lower extremities, cerebellar and sensibility tests revealed normal findings on both sides. No pathological reflexes were registered. The gait was unchanged.

### Diagnostic assessment

**Figure 2:**
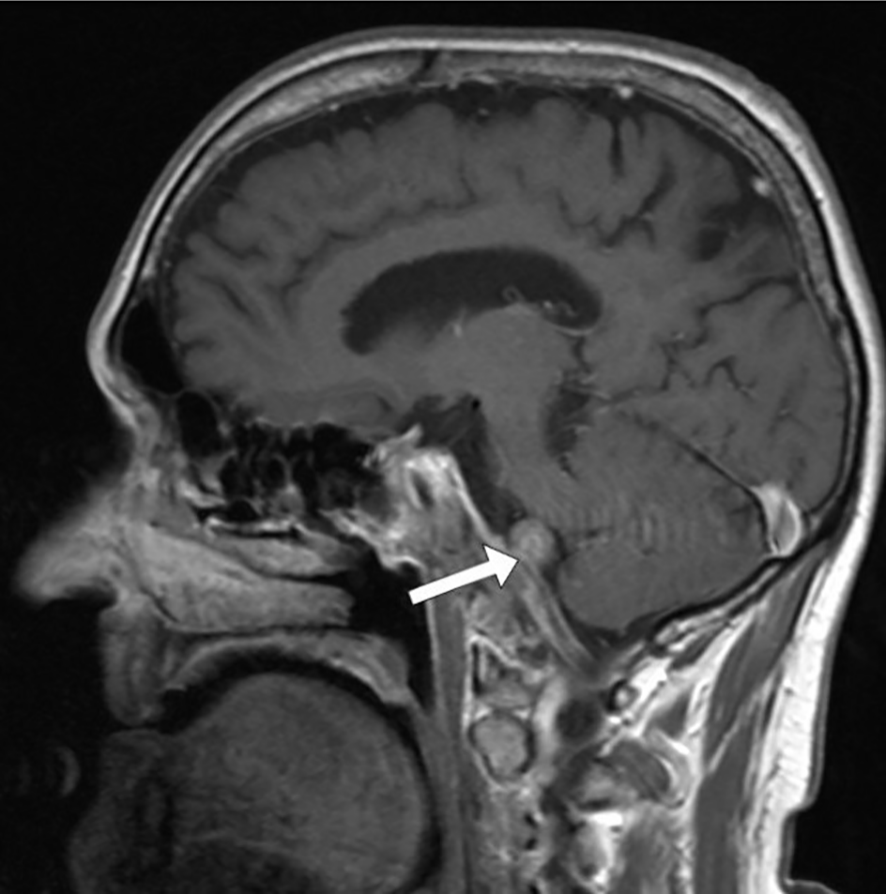
Transverse section in the saggital plane of the endocranium, the arrow points to the meningioma.

**Figure 3:**
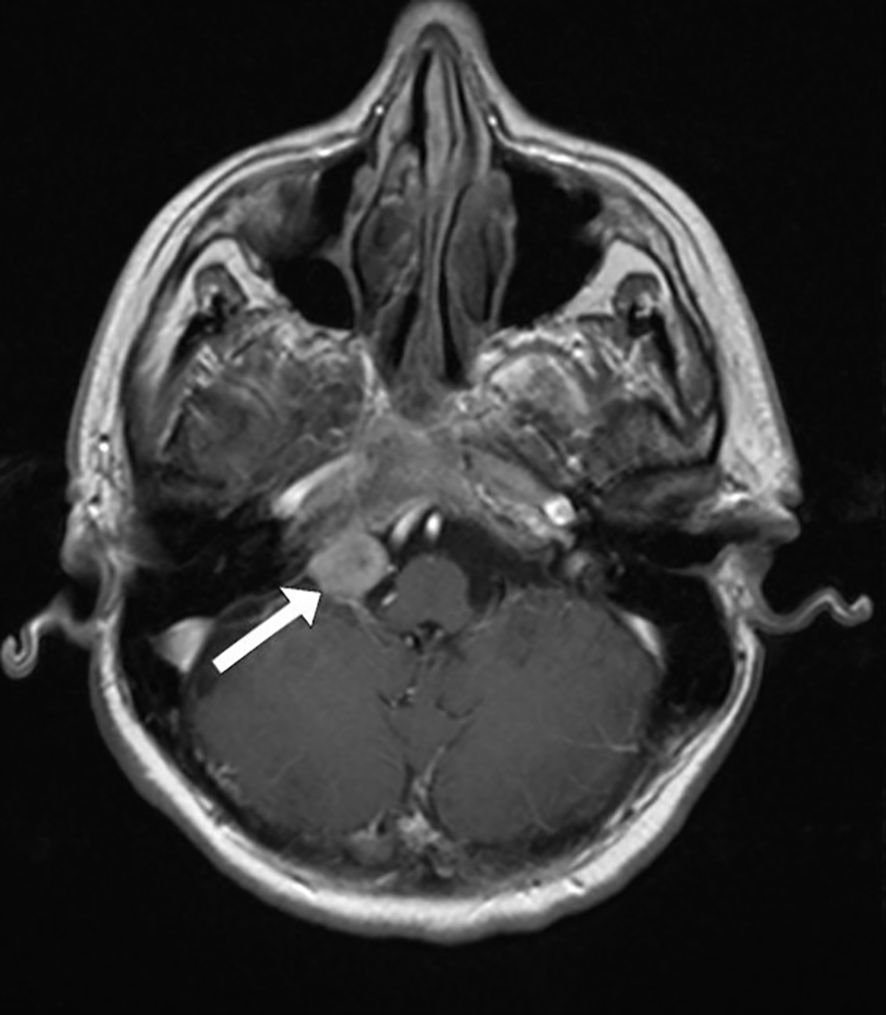
Transverse section in the horizontal plane of the endocranium, the arrow points to the meningioma.

Magnetic resonance imaging (MRI) of the endocranium was performed according to a standard protocol: Extracerebral cerebrospinal fluid spaces are dilated within reductive changes. Chronic microischemic lesions are observed in the periventricular white matter. Mediosagittal structures are neat. Ventricular system is of an orderly size, with adequate content. In the posterior cranial fossa, extraaxial focal lesion is in the right cerebellomedullary cistern, next to the clivus. The change is solid, clearly defined, measuring up to 17x15mm on the axial tomogram (**Figures 2**, **3**). There is a homogeneous, intense post-contrast staining of the described change. There are no signs of descent of the cerebellar tonsils. There are no pathological changes in the orbits. There are no signs of inflammation of the paranasal sinuses and mastoids. Conclusion: Clival meningioma. Bilateral chronic supratentorial microischemic lesions.

### Therapeutic interventions

Neurosurgical intervention was performed using an endoscopic extended transclival approach. Pathohistological and immunohistochemical studies established the diagnosis of meningothelial meningioma grade I according to the World Health Organization (WHO) classification.

### Follow-up and outcomes

The patient tolerated the neurosurgical intervention well. Immediately after surgery, there was no leakage of cerebrospinal fluid and the clinical findings were unchanged. There is right-sided palsy of the hypoglossal nerve with hemiatrophy of the tongue. No postoperative worsening of dysphagia or occurrence of respiratory complications was noted. Considering that the neurosurgical intervention was performed recently, the patient’s follow-up is ongoing.

## Discussion

Meningiomas of the lower clivus are generally accompanied by a disturbance of the function of the lower cranial nerves (4). Usually, at the time of diagnosis of skull base meningioma, patients may complain of gait disturbance, headache, tinnitus, diplopia (5), facial and limb weakness (6), hearing and speech disorders. The initial neurological examination shows the most common signs of cranial nerve dysfunction in the form of facial nerve palsy, acoustic nerve and trigeminal nerve deficits, somewhat less often oculomotor nerve and abducens nerve palsies and glossopharyngeal nerve deficit. Other neurological signs include cerebellar and pyramidal disturbances (3).

We consider our case to be unusual because despite the significant size of the meningioma, the patient only had paralysis of the ipsilateral hypoglossus. By reviewing the available literature, we observed only three cases of patients who also had only hypoglossal nerve paralysis as a clinical sign of clival meningioma. The first case presented is a patient with oral ulcerations, fasciculations and hemiatrophy of the right side of the tongue. A 65-year-old woman was referred for repeated ulcerations on the right side of the tongue preceded by bruising. The ulceration was accompanied by a feeling of discomfort on the right side of the tongue. A physical examination revealed tongue asymmetry due to right-sided hemiatrophy with fasciculations and deviations to the right side. This isolated hypoglossal nerve palsy is due to a meningioma growing in the posterior fossa and displacing the brainstem at the bulbar level (7). The second case is a 52-year-old woman with a large inferior clival meningioma compressing the medulla oblongata, presented with mild difficulty swallowing and right-sided hypoglossal nerve palsy. An endoscopic transnasal extended transclival approach was performed. Residual tumor mass that were strongly adherent on the right side, at the level of the lower cranial nerves, were treated with Gamma Knife surgery. Her symptoms gradually improved after surgery. The patient was followed for 3 years and the tumor was successfully controlled. At the last examination, there were no neurological symptoms (8). The third case is a 72-year-old woman diagnosed with foramen magnum meningioma. She had only progressive paralysis of the hypoglossal nerve and lingual atrophy on the left side. Surgery was performed through a transcondylar approach to expose the hypoglossal canal and completely remove the tumor. Histological examination revealed transitional meningioma. The postoperative course was uneventful. The hypoglossal nerve palsy gradually improved (9).The demographic and clinical characteristics of these three patients with hypoglossal palsy as the only clinical sign caused by meningioma of the clivus are shown in **Table 1**.

**Table 1:** Demographic and clinical characteristics of three patients with hypoglossal palsy as the only clinical sign caused by meningioma of the clivus.

Case	1. Barnadas MA et al. (7)	2. .Kawaguchi A et al. (8)	3. Inaka Y et al. (9)
**Age (years)**	65	52	72
**Gender**	Female	Female	Female
**Symptoms**	A feeling of discomfort, bruising and ulceration of the right side of the tongue, right sided hemiatrophy with fasciculations and tongue deviation to the right	Mild difficulty swallowing, right-sided hemiatrophy of the tongue	Progressive, left sided hemiatrophy and tongue deviation to the left
**Neurosurgical technique**	Not specified	Endoscopic transnasal extended transclival approach Gama Knife surgery	Transcondylar approach
**Histological diagnosis**	Not specified	Not specified	Transitional meningioma
**Complications**	None	None	None
**Follow-up**	Ongoing at time of publication	3 years	Ongoing at time of publication
**Status**	Not specified	No neurological symptoms	Gradually improved

The initial symptom of a meningioma of the lower clivus can be a multi-year progressive headache in the occipital region, with later development of balance impairment and difficulty swallowing (10). A meningioma affecting the lower clivus and foramen magnum caused the patient a headache and numbness of the right upper extremity (11).

We were also interested in other cases of patients who had meningiomas of the clival region accompanied by unusual clinical symptoms and signs at the time of diagnosis.

A review of the existing literature revealed that petroclival meningiomas are rare lesions at the skull base, originating from the upper two-thirds of the clivus, medial to the V-XI cranial nerves. Petroclival meningioma is manifested by trigeminal neuralgia (paroxysmal episodes of facial pain in the distribution of the trigeminal nerve) (12, 13) in less than 5% of cases. It is most often presented in meningioma of tentorial origin with spread over the trigeminal nerve and retrograde invasion into Meckel’s cave (14).

Several cases of petroclival meningiomas with involvement of only the trigeminal nerve have been presented. One patient had only facial numbness in the V2 nerve distribution with normal hearing and facial function (15). The second patient had only left-sided V1, V2 and V3 thermal and painful hypoesthesia, without allodynia (16).

It is thought that pathological laughter (uncontrolled laughter that is disconnected from stimulus and mood) may be an early focal sign of massive compression of the ventrolateral brainstem, as described in case reports of four young and middle-aged patients. Pathological laughter was the only sign from 4 months to 2 years before the enrichment of clinical symptomatology caused by petroclival meningioma. Tumors causing compression of the ventral brainstem must be excluded before the patient is referred to a psychiatrist (17–20).

In accordance with the petroclival position of the meningioma, progressive gait disturbances are possible (21) with disorders of the following cranial nerves: V and VIII cranial nerves with cerebellar ataxia (22), VI cranial nerve with diplopia and homonymous hemianopsia (23), VI cranial nerve with diplopia and trunk ataxia (24), V, VII and VIII cranial nerves with unilateral cervicofacial choreoathetotic dyskinesias (25).

A case of ipsilateral hemifacial spasm caused by a meningioma in the foramen magnum and clivus, which had no direct contact with the VII and VIII cranial nerves, was described (26).

After several months or years of headache and lower cranial nerve dysfunction, limb weakness may develop, in the form of progressive tetraparesis (27). Clinical symptoms of brainstem compression and hydrocephalus (28), with inadequate ventilation and development of respiratory paralysis (29) may be seen during further deterioration of the clinical symptomatology of petroclival meningioma.

The question that intrigued us from the moment we saw our patient’s MRI findings was - how is it possible that a tumor formation of this size is accompanied by an isolated disorder of the function of only one cranial nerve? Was the beginning of tumor proliferation exactly at the level of the right hypoglossal nerve? The fact is that meningiomas grow relatively slowly, giving the nerve tissue the opportunity to gradually “adapt” to compression and to preserve its function up to a certain point. Then, in the later course of the disease, disorders of the functions of the other lower cranial nerves would most likely appear. The patient was referred to us before compression of other lower cranial nerves occurred. Unlike other lower cranial nerves, the supranuclear innervation of the hypoglossal nerve is predominantly from the contralateral cortex. Does that somehow make it more “sensitive” to compression from the outside? If we stay within the framework of theoretical speculations, it could have some significance.

In addition to the wide range of clinical symptoms and signs accompanying meningiomas of the clivus or craniovertebral junction, the differential diagnosis of other pathological changes in the clival area is very important for practical clinical work.

A very interesting retrospective review of nine patients diagnosed with extremely rare pathologies of the craniovertebral junction was recently published. Cases of clival osteoradionecrosis, Ecchordosis Phisaliphora, metastases of hepatocellular carcinoma, capillary hemangioma, embryonal rhabdomyosarcoma, cholesterol granuloma, extradural meningioma, ganglion cyst and histiocytic sarcoma have been described. One of the cases was a patient who complained of intense neck pain. A voluminous extradural mass replacing the left condyle and eroding part of the lower clivus was diagnosed. It was histologically confirmed as meningioma, without relapse after five years (30).

Primary extradural meningiomas in the head constitute 0.8%-1.8% of all meningiomas. The most frequent histopathological subtype was meningothelial meningioma. The prognosis is good in benign cases after complete surgical resections (31).

Ecchordosis Phisaliphora (EP) is a distinct clinical entity defined as a notochordal remnant located on the dorsal surface of the clivus, and occurs in about 2% of autopsies. EP and clival chordoma are different spectra of the same pathology. As these two lesions have completely different prognoses, accurate diagnosis is necessary for proper treatment planning (32). Chordomas and EP can rarely have intracranial hemorrhage (33).

Although they are extremely rare, metastases involving the clivus should be considered in the differential diagnosis with clivus chordoma. A metastatic lesion can be a late and individual expression of the primary tumor (34).

Hepatocellular carcinoma (HCC) is the fifth most common cancer worldwide, and its incidence has been increasing in recent years. Metastases in the nervous system, particularly in the clivus, are rare. The case of a patient with hepatocellular carcinoma treated with transarterial chemoembolization is presented, in which metastasis in the clivus was diagnosed (35).

Capillary hemangiomas are benign vascular lesions involving the skin and soft tissues that usually appear at birth or early in life. Intracranial capillary hemangiomas are exceptionally rare (36).

Skull base cholesterol granulomas are rare lesions that can be accompanied by headache, ipsilateral retroorbital pain, diplopia, ipsilateral blepharospasm and hearing loss. The cases of four patients are presented, only in one the site of the lesion was the clivus (37).

Histiocytic sarcoma is a rare malignant neoplasm of the macrophage-dendritic cell line, which extremely rarely primarily affects the skull base. The case of a patient who had a headache and localized neurological symptoms is presented. Despite the surgical treatment and radiotherapy treatment, the fatal outcome occurred within a few months (38).

## Conclusions

Our case report showed an unusual clinical presentation of clival meningioma. Considering that these tumors are not common in this part of the skull base, attention should be paid to the clinical signs that accompany them. The cause of oligosymptomatic manifestation with peripheral paralysis of the hypoglossal nerve was precisely defined using magnetic resonance imaging. This case highlights the importance of timely and comprehensive differential diagnosis of unusual clinical manifestations. Early diagnosis of clivus meningioma is important both for improving the prognosis and avoiding neurological consequences.

## Data availability statement

The raw data supporting the conclusions of this article will be made available by the authors, without undue reservation.

## Ethics statement

Written informed consent was obtained from the individual(s) for the publication of any potentially identifiable images or data included in this article.

## Author contributions

JS: Writing – original draft. BŽ: Writing – review & editing. VĐ: Writing – review & editing.
